# Viromes of Freshwater Fish with Lacustrine and Diadromous Life Histories Differ in Composition

**DOI:** 10.3390/v14020257

**Published:** 2022-01-27

**Authors:** Benjamin J. Perry, Mitra Mohamadi Darestani, Motia Gulshan Ara, Amélie Hoste, Jennifer M. Jandt, Ludovic Dutoit, Edward C. Holmes, Travis Ingram, Jemma L. Geoghegan

**Affiliations:** 1 Department of Microbiology and Immunology, University of Otago, Dunedin 9016, New Zealand; benjamin.perry@otago.ac.nz; 2 Department of Zoology, University of Otago, Dunedin 9016, New Zealand; mohmi699@student.otago.ac.nz (M.M.D.); motia.ara@postgrad.otago.ac.nz (M.G.A.); amelie.hoste@wanadoo.fr (A.H.); jenny.jandt@otago.ac.nz (J.M.J.); ludovic.dutoit@otago.ac.nz (L.D.); travis.ingram@otago.ac.nz (T.I.); 3 Sydney Institute for Infectious Diseases, School of Life and Environmental Sciences and School of Medical Sciences, The University of Sydney, Sydney 2006, Australia; edward.holmes@sydney.edu.au; 4 Institute of Environmental Science and Research, Wellington 5022, New Zealand

**Keywords:** virus, fish, diadromous, lacustrine, ecology, meta-transcriptomics

## Abstract

Viruses that infect fish are understudied, yet they provide important evolutionary context to the viruses that infect terrestrial vertebrates. We surveyed gill tissue meta-transcriptomes collected from two species of native freshwater fish from Aotearoa New Zealand—*Retropinna retropinna* and *Gobiomorphus cotidianus*. A total of 64 fish were used for gill tissue meta-transcriptomic sequencing, from populations with contrasting life histories—landlocked (i.e., lacustrine) and diadromous—on the South Island and Chatham Islands. We observed that both viral richness and taxonomic diversity were significantly associated with life history and host species, with lacustrine *R. retropinna* characterised by higher viral alpha diversity than diadromous *R. retropinna*. Additionally, we observed transcripts of fish viruses from 12 vertebrate host-associated virus families, and phylogenetically placed eight novel RNA viruses and three novel DNA viruses in the *Astroviridae*, *Paramyxoviridae*, *Orthomyxoviridae*, *Rhabdoviridae*, *Totiviridae*, *Poxviridae*, *Alloherpesviridae,* and *Adintoviridae* in their evolutionary contexts. These results represent an important survey of the viruses that infect two widespread native fish species in New Zealand, and provide insight useful for future fish virus surveys.

## 1. Introduction

Fish represent basal evolutionary lineages to all extant terrestrial vertebrate species [1]. Like all organisms, fish are susceptible to viral infection and have likely co-diverged with many viral groups over long evolutionary timescales. Accordingly, revealing the viruses that infect fish species is useful for understanding the timescale of virus evolution and the relative frequency of virus–host co-divergence versus cross-species virus transmission [2], and provides useful evolutionary context to the viruses that infect higher vertebrates [3].

Aotearoa New Zealand separated from the Gondwanan supercontinent ca. 85–82 million years ago as part of the subcontinent Zealandia—known as Te Riu-a-Māui in Te Reo Māori [4,5]. The Zealandia continental *waka* carried unique Gondwanan species into ecological isolation, and during this process, early mammalian lineages became extinct [6]. This unique ecological isolation resulted in many endemic and native species of insects, birds, reptiles, and fish [7,8,9,10]. Analysis of the viruses that infect these endemic species provides an opportunity to identify whether this vicariance event has left an impression on virus ecology and evolution.

*Retropinna retropinna* (common smelt) and *Gobiomorphus cotidianus* (common bully) are two widespread species of native New Zealand fish. Both are facultatively diadromous, normally spawning in freshwater and completing at least part of their life cycle in the sea, but they are also capable of living entirely in freshwater when they become landlocked [11,12]. *R. retropinna* is a pelagic fish species found throughout lowland rivers, estuaries, and coastal lakes of New Zealand, including Rēkohu (Chatham Island) [13]. *G. cotidianus* is a benthic fish species found in rivers and estuaries across the North and South Islands of New Zealand, as well as in isolated freshwater lake populations [11]. The niches occupied by these fish, their ubiquitous distribution across New Zealand, and their ability to live in both fresh and salt water makes them interesting models to understand how ecological factors and life history traits may influence the composition and abundance of fish viruses.

The objective of this study was to better understand the determinants of virus ecology in diadromous and lacustrine populations of two New Zealand endemic fish species. Using meta-transcriptomics, we surveyed the viromes of *R. retropinna* and *G. cotidianus* individuals collected from lacustrine and diadromous populations from the South Island and Rēkohu, Aotearoa New Zealand. In doing so, we identified multiple novel RNA and DNA viruses, and determined whether viral ecology differed among lacustrine and diadromous fish hosts.

## 2. Materials and Methods

### 2.1. Iwi Consultation and Animal Ethics Approval

The use of native species of Aotearoa New Zealand for research was approved through iwi/imi consultation with the Ngāi Tahu Research Consultation Committee (University of Otago) and the Hokotehi Moriori Trust (Chatham Island). Animal ethics approval was obtained from the University of Otago Animal Ethics Committee (Approval Number: AUP-19-196).

### 2.2. Fish Gill Meta-Transcriptomic Sequencing

**Figure 1:**
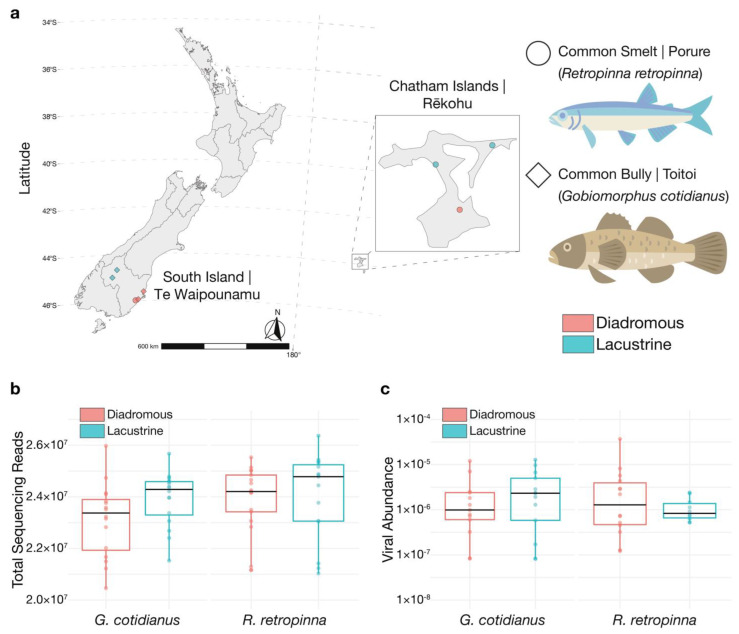
(**a**) Sampling locations for *R. retropinna* and *G. cotidianus* collected on the South Island and Chatham Islands of New Zealand. Fish life-history is indicated as diadromous or lacustrine. (**b**) Total paired-end sequencing reads for gill-tissue meta-transcriptome libraries. (**c**) Standardised abundance estimates of viral transcripts identified in gill-tissue meta-transcriptome sequencing libraries. Fish illustrations by Hamish Thompson.

Adult individuals of each fish species were collected from four source populations, two diadromous and two lacustrine, during December 2019–January 2020. Diadromous *R. retropinna* were collected from the Taieri River (eight fish; 45°59′33.3″ S 170°09′03.6″ E) on the South Island and Te Awainanga River (nine fish; 43°59′18.2″ S 176°27′09.8″ W) on Rēkohu, and lacustrine *R. retropinna* were collected from Lake Rotorua (eight fish; 43°45′35.8″ S 176°17′16.2″ W) and Tennants Lake (seven fish; 43°49′25.0″ S 176°34′05.0″ W), both on Rēkohu (Figure 1a). Diadromous *G. cotidianus* were collected from Waikouaiti River (eight fish; 45°36′29.7″ S 170°37′22.5″ E) and Otokaia Creek (eight fish; 45°56′42.5″ S 170°19′14.7″ E), and lacustrine *G. cotidianus* were collected from Lake Hayes (eight fish; 44°58′08.7″ S 168°48′51.4″ E) and Lake Wanaka (eight fish; 44°40′11.7″ S 169°01′57.2″ E) (Figure 1a).

In total, 64 fish were captured using fyke nets or seine nets and transported to an indoor laboratory space within one hour of collection. Samples were collected as part of a larger study to examine gene expression changes in response to fresh and salt water, rather than specifically for virome analysis. For this reason, four fish were individually placed in 4 L containers of freshwater from their site of collection. Another four fish from the same cohort were gradually transferred to 4 L containers of 30 parts per thousand salt water made by combining source water with artificial sea salt (Instant Ocean, Blacksburg, VA, USA). Fish were housed in fresh or salt water with aeration for 12 h prior to sacrifice. Fish were sacrificed with AQUI-S 20 E (AQUI-S New Zealand Ltd., Lower Hutt, New Zealand), length and weight measurements were taken, and gill tissue was then immediately excised, placed in RNA stabilisation solution (RNALater; Qiagen, Hilden, Germany), and frozen at –80 °C within one week. Tissue was stored at −80 °C until RNA was extracted.

RNA was extracted from frozen tissue samples using the RNeasy Mini Kit (Qiagen). Total RNA was shipped in Bio-Bottles (Auckland, New Zealand) on dry ice to Novogene (Singapore) where RNA sequencing libraries were prepared with poly-A enrichment to increase the proportion of mRNA, and indexed and pooled for 150 bp paired-end sequencing on an Illumina NovaSeq 6000.

### 2.3. Virome Assembly and Virus Identification

Virome assembly and analysis used a previously described analysis pipeline [14]. Briefly, paired reads were trimmed and de novo assembled using Trinity v2.11 [15]. Assembled transcripts were annotated with BLASTn using the NCBI nucleotide (nt) database [16], Diamond (BLASTx) [17] using the NCBI non-redundant protein database (nr), and palmscan for identifying RNA-dependent RNA polymerase (RdRp) motifs [18]. A BLAST e-value threshold of 1 × 10^−5^ was used for filtering viral transcripts, and a palmscan -*hiconf* high confidence threshold was used for filtering the identified RdRps. Putative viral transcripts identified in this way were then assigned to vertebrate (i.e., fish)-associated viruses or non-vertebrate (non-fish) viruses based on the best BLASTn/Diamond matches, as well as the associated taxonomic information in the NCBI taxonomy database [19] in conjunction with Virus-Host DB [20]. In instances where the likely viral host was not discernible from the metadata of homologs, phylogenetic analysis with a selection of similar viruses from the NCBI RefSeq database was used to infer the most likely host of the virus (Figure S1).

Viral transcript abundances were estimated using the Trinity implementation of RNA-Seq by Expectation Maximization (RSEM) [21]. Viral transcripts with expected counts of less than 0.1% of the highest abundance sample for that viral family were removed to mitigate the effect of index hopping, as previously described [14]. Viral transcripts identified in this way were compiled and those with translatable reading frames suitable for phylogenetic analysis were further analysed for their evolutionary relationship to similar viruses. Abundance estimates were compiled in a taxa abundance table and used in ecological analysis of viromes. Virus abundance estimates were summarised at viral family level and standardised as a proportion of the sequencing reads in each library. Only viral transcripts identified from eukaryotic-associated viruses were analysed. For heatmap presentation of viral abundances, the total estimated abundance of viral transcripts for a given viral family was standardised by the sample library size, and then the standardised viral abundances were normalised across samples by calculating their proportions of viral transcripts observed in a given viral family.

### 2.4. Virus Phylogenetic Analysis

Translated viral contigs protein sequences of RdRp or DNA-dependent DNA polymerase (DdDp) were aligned with homologous representative protein sequences from viruses within the same viral family using MAFFT v7.450 (L-INS-I algorithm) [22]. Amino acid sequence alignments were trimmed of taxonomically uninformative regions using trimAl v1.2 (-gt 0.9-cons 60) [23] prior to phylogenetic analysis with IQ-TREE using 1000 replicates of ultra-fast bootstrapping (-bb 1000) and the default amino acid substitution model (-m LG) [24,25]. The resulting trees were rooted at their midpoints and sorted by increasing branch length.

### 2.5. Ecological Analysis of Virome Alpha and Beta Diversity

Data handling and plotting generally used R (4.0.3) and the tidyverse [26]. Sampling location plots were made with ggplot (implemented in the tidyverse) and the sf [27], ggspatial [28], nzcensr [29] packages. Normalised virus family abundance values were used to make heatmaps of viral abundance using the heatmaply R package [30] using the “ward.D” clustering algorithm implemented in hclust. Statistical analysis of virus abundance and diversity used the vegan [31], pscl [32,33], statmod [34], and tweedie [35,36,37] packages. Alpha diversity was measured using observed richness as well as the inverse Simpson index, which approximates the richness of an evenly abundant community needed to achieve the level of diversity present in a sample virome [38].

Statistical comparison of observed richness and an inverse Simpson index between life-history types (lacustrine versus diadromous) were stratified by fish species and modelled using generalised linear models (glm). For observed richness, hurdled glms were used that first tested the frequency of observing viral transcripts using a binomial family distribution, and secondly modelled the effect on observed richness in samples with richness > 0 using a Poisson family distribution. To test for effects of life history and species on an inverse Simpson index, Tweedie family glms were used to deal with the zero-inflated nature of the data, and used a gamma family distribution for generalisation. In both cases, log link functions were used to relate the generalised distributions to the linear predictors.

Compositions of vertebrate-associated viral communities were compared using multivariate ordination methods. Analysis of homoscedasticity between test groupings was done using a Bray–Curtis distance matrix modelled in the betadisper function in vegan and tested with permutational tests for grouping species and life history, and the Tukey honest significant differences test for the source. To test for effects of species, life-history type, and location on viral composition, we used permutational multivariate analysis of variance with the adonis2 function (implemented in vegan) using the factor species, life history, and source in an NMDS model of Bray–Curtis dissimilarity of standardised abundance. Ordination plotting of standardised abundance used constrained correspondence analysis, also with species, life history, and location. ANOVA-like permutation analysis of the CCA model was used to corroborate the significance of the species, life history, and source as constraints. Ordination plots were generated using the ggord package [39].

We also determined whether the fresh and salt water treatment that the fish were exposed to post-capture had any effect on virome composition following the above methodology.

### 2.6. Viral Nomenclature

Following consultation, novel viruses identified in *R. retropinna* samples collected from Rēkohu/Chatham Island were named using a ta rē Moriori name, porure. Novel viruses identified in the *G. cotidianus* samples collected from the South Island were named using a Te Reo Māori name for *G. cotidianus*, Toitoi.

## 3. Results

In total, 32 *R. retropinna* and *32 G. cotidianus* were collected from two lacustrine and two diadromous locations each from across the South Island of New Zealand and Chatham Islands (Figure 1a). Meta-transcriptomic sequencing of gill-tissue-derived RNA yielded between 20 and 26 million 2 × 125 bp paired-end sequencing reads (40–52 million single reads) per sample, with no significant difference in yield between species or lacustrine and diadromous fish (Figure 1b). No significant effects on standardised viral abundance were identified for fish species or life history (Figure 1c). RPS13 is a constitutively expressed ribosomal subunit in fish cells, and hence can be used as a marker of host gene abundance. The standardized abundance of RPS13 transcripts differed significantly between species and lacustrine and diadromous *R. retropinna,* but not *G. cotidianus* (Figure S2).

### 3.1. Novel Fish Viruses

Among the viruses discovered here, we found several transcripts containing the RdRp or DdDp, enabling us to conduct phylogenetic analysis and describe novel viral lineages. 

#### 3.1.1. *Astroviridae*

**Figure 2:**
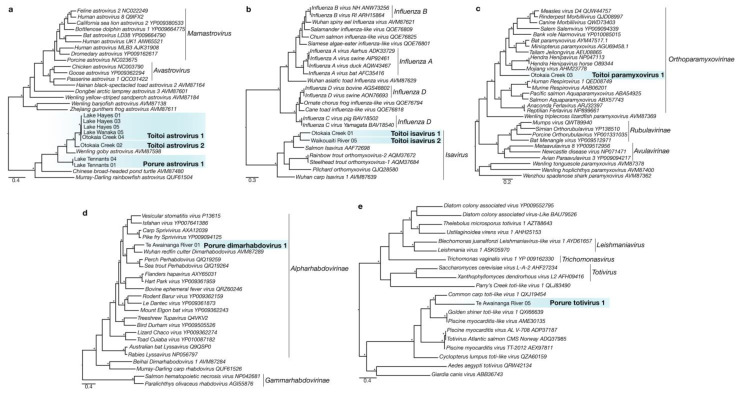
Maximum likelihood phylogenetic trees of the RNA-dependent RNA polymerase from representative viruses from the (**a**) *Astroviridae*, (**b**) *Orthomyxoviridae*, (**c**) *Paramyxoviridae*, (**d**) *Rhabdoviridae*, and (**e**) *Totiviridae*. Lineages corresponding to novel fish viruses described in this study are highlighted in blue. Phylogenetic branches with significant bootstrap values indicated with asterisks (>70). Branches are scaled to the number of amino acid substitutions per site. Amino acid length of novel RdRp sequences identified in this study used for alignment can be found in Table S1.

We identified a novel positive-sense single-stranded RNA (ssRNA) virus in the *Astroviridae* family in the gill meta-transcriptomes of *R. retropinna*. This virus, named Porure astrovirus 1, falls within a clade of astroviruses sampled from fish species throughout the Asia-Pacific region (Figure 2a), which included two additional novel astroviruses from *G. cotidianus* (this work). Porure astrovirus 1 RdRp transcripts were identified in two *R. retropinna* gill meta-transcriptomes collected from Tennants Lake on the Chatham Islands (Table S1). The two transcripts (99.7% amino acid identity) were both greater than 1000 bp in length and contained the conserved RdRp motif ‘YGGD’ found in many RNA viruses [40]. The relative abundance of *Astroviridae* transcripts was 0.00018% and 0.00017% for Tennants Lake 04 and 01, respectively (Table S2). The most similar astrovirus RdRp in the NCBI non-redundant protein database to Porure astrovirus 1 was *Hainan astro-like virus 2* (50.53% amino acid identity; e-value = 9 × 10^−1^^24^); however, the host for this virus is unknown. The most similar astrovirus with a host association was *Wenling gobies astrovirus* (52.35% amino acid identity; e-value = 3 × 10^−1^^21^) [2].

We identified two novel species of the astrovirus in the *G. cotidianus* gill meta-transcriptomes and have named these Toitoi astrovirus 1 and Toitoi astrovirus 2 (Figure 2a). Toitoi astrovirus 1 was identified in individuals sampled from the lacustrine populations in Lake Hayes and Lake Wanaka, as well as the diadromous population in Otokaia Creek (Figure 2a). In one of the Lake Hayes samples, we recovered a 7045 bp transcript of Toitoi astrovirus 1, which contained a truncated Orf1a open reading frame, and full-length open reading frames for Orf1b and Orf2 proteins, followed by a 295 bp putative 3′ untranslated region. This arrangement of Toitoi asrovirus 1 genes is similar to other known astroviruses [41]. Toitoi astrovirus 2 was identified in an individual captured at Otokaia Creek. The RdRp domain from Toitoi astrovirus 2 shared 78.2% ammino acid identity across the 234 amino acid regions of the RdRp recovered with Toitoi astrovirus 1; indicating the two viruses were distinct species. The most closely related astrovirus to Toitoi astrovirus 1 and Toitoi astrovirus 2 was *Wenling gobies astrovirus* [2] that shared 64.5% and 78.2% amino acid identity across the commonly aligned regions, respectively (Table S1).

#### 3.1.2. *Orthomyxoviridae*

Two novel viruses from the *Orthomyxoviridae* were identified in *G. cotidianus* collected from the diadromous populations at Otakaia Creek and Waikoutaiti River, and named Toitoi isavirus 1 and Toitoi isavirus 2 (Figure 2b). The Toitoi isavirus 1 PB1 transcript was 1038 bp in length, and the Toitoi isavirus 2 transcript, 443 bp (Table S1). Relative abundances of *Orthomyxoviridae* for the samples containing Toitoi isavirus 1 and Toitoi isavirus 2 were 0.00014% and 0.00022% of sequences, respectively (Table S2). Phylogenetic analysis of the PB1 RdRp indicated that both viruses were within the *Isavirus* genus, which contains other orthomyxoviruses from fish (Figure 2b). Toitoi isavirus 1 and Toitoi isavirus 2 PB1 proteins shared 32.3% amino acid identity across the 128 residues of PB1 sequence common to the two transcripts. The top BLAST result of Toitoi isavirus 1 and Toitoi isavirus 2 PB1 proteins was *Salmon isavirus* PB1 (ACJ37394.1) [42], sharing 51.4% and 34.3% amino acid identity, respectively.

#### 3.1.3. *Paramyxoviridae*

A novel negative-sense ssRNA virus in the *Paramyxoviridae* named Toitoi paramyxovirus 1 was identified in a diadromous *G. cotidianus* collected in Otokaia Creek. Two transcripts of the virus were recovered with a cumulative length of 13,179 bp and had the highest relative abundance of any vertebrate virus at 0.011% (Table S2). These transcripts encoded open reading frames for the N, P, M, and F proteins in addition to a fragment of the L protein containing the RdRp (Table S1). These proteins were in an arrangement similar to that of other paramyxoviruses. The top BLAST result to the Toitoi paramyxovirus 1 L protein was *Bat paramyxovirus* (AYM47517.1), sharing 52.76% amino acid identity (e-value = 1.0 × 10^−1^^72^) (Table S1). Phylogenetic analysis placed Toitoi paramyxovirus 1 as a basal lineage to the *Morbillivirus*, *Salemvirus*, *Narmovirus*, and *Henipavirus* genera—the first known fish virus identified in this evolutionary position (Figure 2c).

#### 3.1.4. *Rhabdoviridae*

A novel negative-sense ssRNA virus in the *Rhabdoviridae* family, termed Porure dimarhabodovirus 1, was identified in a *R. retropinna* sampled from a diadromous population in the Te Awainanga River on Chatham Island (Figure 2d). We recovered a 525 bp transcript of the RdRp from this sample, which contained *Rhabdoviridae* transcripts at a relative abundance of 0.00018% (Table S2). Phylogenetic analysis of the translated RdRP containing transcript indicated that Porure dimarhabodovirus 1 was in a clade of fish dimarhabodovirus that included *Wuhan redfin culter dimarhabodovirus* [2]; the two viruses shared an amino acid identity of 62.3%, indicating they were separate species (Table S1).

#### 3.1.5. *Totiviridae*

A novel double-stranded RNA (dsRNA) virus in the *Totiviridae,* named Porure totivirus 1, was described from a *R. retropinna* individual sampled from the diadromous population in Te Awainanga River (Table S1; Figure 2e). Transcripts of *Totiviridae* origin were present at a relative abundance of 0.00000048% in the gill-tissue meta-transcriptome of this individual (Table S2). We identified a 435 bp transcript corresponding to a region of the Porure totivirus 1 RdRP, and phylogenetic analysis of the amino acid alignment revealed that Porure totivirus 1 was in a clade of other fish totiviruses (Table S1; Figure 2e). The top BLAST results to the Porure totivirus 1 RdRp fragment recovered was *Common carp toti-like virus 1* at 42.66% (e-value = 2 × 10^−2^^5^) amino acid identity, indicating Porure totivirus 1 was a novel totivirus species (Table S1).

#### 3.1.6. *Poxviridae*

**Figure 3:**
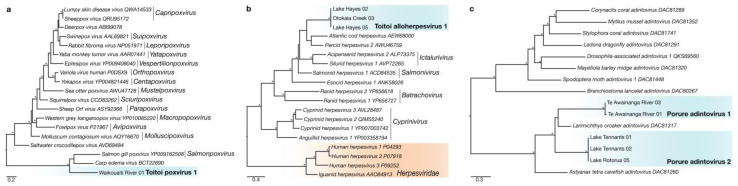
(**a**) Maximum-likelihood phylogenetic tree of the Rpo18 subunit of the DNA-dependant RNA polymerase from representative viruses across the *Poxviridae*. (**b**) Maximum-likelihood phylogenetic tree of the DNA-dependant DNA polymerase from representative virus from the *Alloherpesviridae* with the *Herpesviridae* (indicated in orange) as an outgroup. (**c**) Maximum-likelihood phylogenetic tree of DNA polymerase beta protein homologues from representative viruses across the *Adintoviridae*. Lineages corresponding to novel fish viruses described in this study highlighted in blue. Asterisks indicated phylogenetic lineages with significant bootstrap values (>70). Branches are scaled to the number of amino acid substitutions per site. Amino acid length of novel DdDp proteins identified in this study used for alignment can be found in Table S1.

A novel fish poxvirus, termed Toitoi poxvirus 1, was identified in the gill meta-transcriptome of a *G. cotidianus* fish collected from Waikouaiti River (Figure 3a). The relative abundance of *Poxviridae* transcript in the sample containing Toitoi poxvirus 1 was 0.00000014% of reads (Table S2). A 256 bp transcript encoding a region of the Rpo18 subunit of the DdDp of Toitoi poxvirus 1 was identified (Table S1). The best BLAST result to the Toitoi poxvirus 1 Rpo18 subunit was *Carp edema virus* (BCT22690.1) [43] at 69.0% amino acid similarity. Phylogenetic analysis of Toitoi poxvirus 1 with other known poxviruses indicated that the Toitoi poxvirus 1 was part of what appeared to be a fish-specific clade of poxviruses that was highly divergent from other known vertebrate poxviruses (Figure 3a).

#### 3.1.7. *Alloherpesviridae*

Transcripts from viruses in the *Alloherpesviridae* were identified in *G. cotidianus* fish from the lacustrine population in Lake Hayes and the diadromous population in Otokaia Creek, ranging in relative abundances from 0.00000008% to 0.00000048% of reads (Table S2). Three transcripts (two from Lake Hayes, one from Otokaia Creek) were identified which encoded for a region of the DdDp, ranging in length from 230 bp to 391 bp (Table S1). The best BLAST results to these protein fragments in the NCBInr database were to the DdDp of *Percid herpesvirus 2* (AWU46759.1) [44], and ranged in amino acid similarity from 47–55%, indicative of a novel viral species. This novel fish alloherpesvirus was named Toitoi alloherpesvirus 1 (Table S1). Phylogenetic analysis of Toitoi alloherpesvirus 1 DdDp indicated that it formed a unique lineage within a large clade of fish viruses (Figure 3b).

#### 3.1.8. *Adintoviridae*

Transcripts from two novel *Adintoviridae* viruses were identified in *R. retropinna* fish captured from Lake Tennants, Lake Rotorua, and Te Awainanga River on Rēkohu (Figure 1a). These transcripts ranged in standardised abundance from 0.000000081% to 0.00000022% across the six individuals they were identified from (Table S2). Five of these individuals (two from Lake Tennants, one from Lake Rotorua, and one from Te Awainanga River) had transcript encoding for fragments of the PolB protein, ranging in size from 57–155 amino acids in length (Table S1). Phylogenetic analysis of the PolB fragments with other adinotviruses indicated that two species of Porure adintovirus were present (Figure 3c). The top BLAST result to all fragments was the PolB protein of *Larimichthys croaker adintovirus* (DAC81317), ranging from 48.2% to 73.6% amino acid identity (Table S1).

### 3.2. Viral Abundance and Diversity

**Figure 4:**
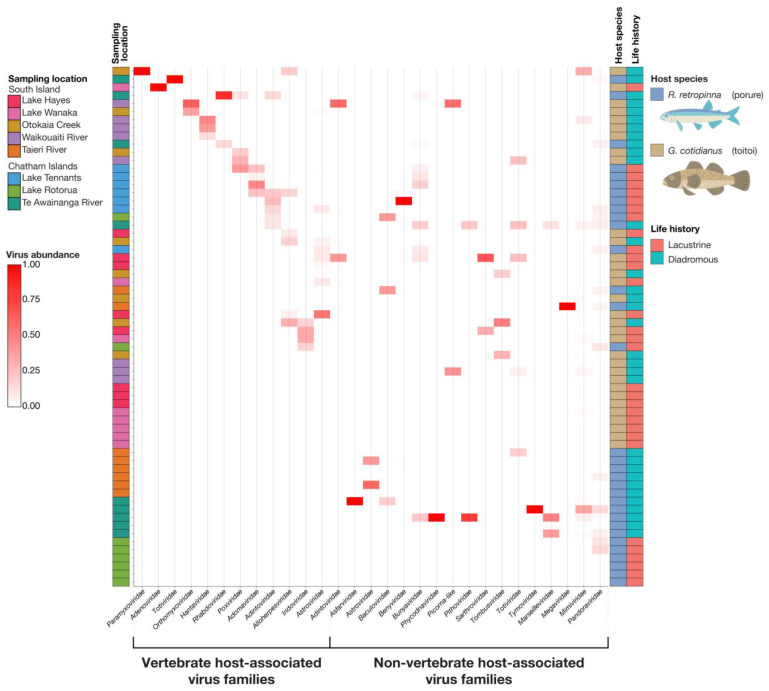
Heatmap of normalised abundance of viral transcripts within viral families, identified in the gill-tissue meta-transcriptomes of diadromous and lacustrine *R. retropinna* and *G. cotidianus*. Host associations were inferred from sequence homology with previously described viruses. Samples were clustered using the ward.D method of the hclust function.

**Figure 5:**
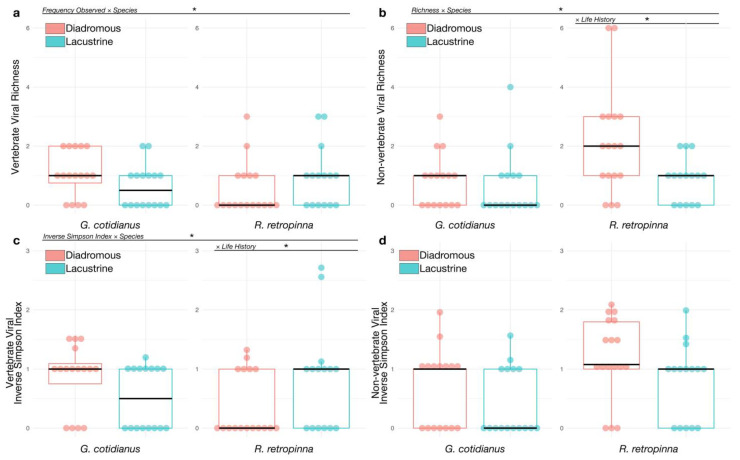
(**a**) Vertebrate host-associated viral richness observed in *R. retropinna* and *G. cotidianus* gill-tissue meta-transcriptomes. (**b**) Non-vertebrate host-associated viral richness observed in *R. retropinna* and *G. cotidianus* gill-tissue meta-transcriptomes. Viral richness was modelled using fish species and life history with hurdled generalised linear models which modelled the frequency of viral presence and subsequently sample richness for those samples with viruses present. (**c**) Inverse Simpson index of vertebrate host-associated virus diversity in *R. retropinna* and *G. cotidianus* gill-tissue meta-transcriptomes. (**d**) Inverse Simpson index of non-vertebrate host-associated virus diversity in *R. retropinna* and *G. cotidianus* gill-tissue meta-transcriptomes. Inverse Simpson index was modelled using species and life history with Tweedie family generalised linear models for zero-inflated continuous data. Asterisks indicate significant effects at *p* < 0.05.

A summary of normalised viral abundances for viral transcripts identified across the gill-tissue meta-transcriptomes of the 64 fish sampled in this study is presented in Figure 4. Statistical analysis of the patterns of viral abundance and diversity related to host biology and ecology did identify a number of significant effects. Fish species did not have a significant association with vertebrate host-associated viral richness (z = 0.237; *p* = 0.813). The mean vertebrate host-associated viral richness was 1.53 (±0.834 SD) in *R. retropinna*, and 1.35 (±0.489 SD) in *G. cotidianus*. Fish species did have a significant association with the frequency of identifying vertebrate host-associated viral transcripts (z = −2.218; *p* = 0.023) (Figure 5a), with vertebrate host-associated viral transcripts observed in 62.5% of *G. cotidianus* and 46.9% of *R. retropinna* samples (Figure 4). Fish species also had a significant association with non-vertebrate host-associated viral richness (z = 2.112; *p* = 0.035) in *R. retropinna* (2.04 ± 1.43 SD) and *G. cotidianus* (1.53 ± 0.915 SD) gill-tissue meta-transcriptomes (Figure 5b). Non-vertebrate host-associated viral transcripts were identified in 75.0% of *R. retropinna* and 46.9% of *G. cotidianus*. A significant association with the Inverse Simpson index of vertebrate host-associated viruses (t = −2.168; *p* = 0.0342) was also observed for fish species (Figure 5c). No significant effect of species on the inverse Simpson index was observed for non-vertebrate host-associated viruses (z = 1.725; *p* = 0.0897) (Figure 5d). Spearman’s rank correlation test of vertebrate-associated viral alpha diversity with Fulton’s condition factor (weight × length^3^ × 100,000) [45] indicated there was no significant correlation within either lacustrine of diadromous populations (Figure S3).

Life history did not have a significant association with viral richness. Specifically, 54.5% of diadromous fish and 54.8% of lacustrine had vertebrate host-associated viral transcripts, and 69.7% diadromous and 51.6% of lacustrine had non-vertebrate host-associated viral transcripts. When stratified by fish species, life history had a significant association with the *R. retropinna* inverse Simpson index for vertebrate host-associated viruses (t = 2.007; *p* = 0.0493) (Figure 5c), with lacustrine individuals having higher average vertebrate host-associated viral richness (Figure 5c). Conversely, when controlling for fish species, life history had a significant association with richness of non-vertebrate host-associated viruses identified in gill-tissue meta-transcriptomes of *R. retropinna* (z = −2.450; *p* = 0.0143), with lacustrine *R. retropinna* having lower richness than diadromous (Figure 5b). No significant differences in the inverse Simpson index for vertebrate- or non-vertebrate-associated viruses was observed between diadromous and lacustrine *G. cotidianus*.

**Figure 6:**
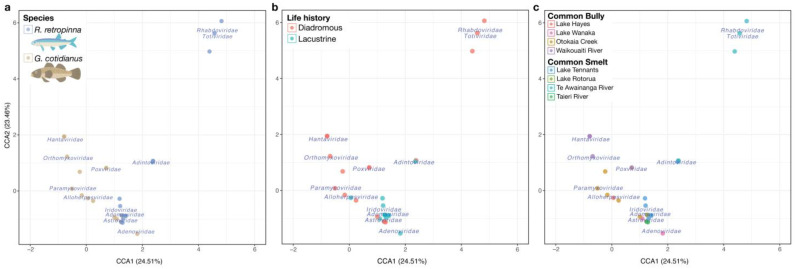
(**a**) Constrained correspondence analysis of vertebrate-associated viral diversity with sample fish species indicated in blue for *R. retropinna* and beige for *G. cotidianus*. (**b**) Constrained correspondence analysis of vertebrate-associated viral diversity with fish life history indicated in pink for diadromous fish and blue for lacustrine fish. (**c**) Constrained correspondence analysis of vertebrate-associated viral diversity with fish source population indicated. Viral taxonomic family centroids indicated in blue text.

The effect of species, life history, and source on vertebrate-associated virome composition was analysed using permutational multivariate analysis of variance using distance matrices, implemented in the vegan adonis2 function, using Bray–Curtis dissimilarity in an NMDS model. A test of homoscedasticity in the dispersion of samples within test groupings identified no significant difference in the variation of species groups (F = 1.67, *p* = 0.203) or life history groups (F = 0.55, *p* = 0.467), but did identify a significant difference in the dispersion of samples, with the Tairie source group being significantly different in dispersion from all other sources at *p* < 0.05. The adonis2 test indicated that species (R^2^ = 0.05032; *p* = 0.0128), life history (R^2^ = 0.05535; *p* = 0.0062), and source (i.e., sampling location) (R^2^ = 0.18481; *p* = 0.0142) had significant associations with beta diversity. Ordination of vertebrate-associated viromes using constrained correspondence analysis (CCA) (Figure 6), and a permutation test of the CCA terms corroborated the adonis2 test results (species, F = 3.1543, *p* = 0.001; life history, F = 4.6915, *p* = 0.001; source, F = 2.5307, *p* = 0.001). The CCA ordination plots illustrated the significant effects of the factor species (Figure 6a), life history (Figure 6b), and source (Figure 6c), as *R. retropinna* and *G. cotidianus* individuals generally separated from one another on this basis.

Sampling location (source) had the strongest influence on virome composition according to the adonis2 analysis, which can be observed in the CCA ordination of sample betadiversity (Figure 6c). Diadromous *R. retropinna* samples from Te Awainanga River clustered in proximity to the *Totiviridae* and *Rhabdoviridae* centroids. The diadromous *G. cotidianus* sources, Otokaia Creek and Waikouaiti River, were distinct from the lacustrine *G. cotidianus* collected from Lake Hayes and Lake Wanaka. The Waikouaiti River individuals clustered near the *Hantaviridae* and *Orthomyxoviridae* centroids, while the Otokaia Creek samples clustered near the *Paramyxoviridae* centroid. The *Astroviridae* centroid was clustered with the Lake Hayes, Lake Wanaka, Otokaia Creek, and Lake Tennants samples, but not Waikouaiti Creek or Te Awainanga River, representative of the frequency of *Astoroviridae* viral transcripts identified within the fish gill-tissue meta-transcriptomes (Figure 6c). In general, the CCA ordination plots revealed similar trends to the statically significant effects indicated by the adonis2 and ANOVA analysis of species, life history, and source on vertebrate-associated viral beta diversity.

Importantly, we found no association between fresh and salt water treatment on vertebrate- or non-vertebrate-associated virus, inverse Simpson index, beta diversity, nor on viral transcript abundance.

## 4. Discussion

We surveyed the viromes of two fish species from across Aotearoa New Zealand to identify novel viruses infecting these native species while exploring the ecological factors that influence viral composition and diversity. In our study of *R. retropinna* and *G. cotidianus* gill-tissue meta-transcriptomes, we phylogenetically placed eight novel fish RNA viruses (Figure 2) and three novel fish DNA viruses (Figure 3) from eight viral families: *Astroviridae*, *Paramyxoviridae*, *Orthomyxoviridae*, *Rhabdoviridae*, *Totiviridae*, *Poxviridae*, *Alloherpesviridae,* and *Adintoviridae* (Table S1) in their respective evolutionary contexts. The recovery and analysis of viral transcripts from meta-transcriptome data provides useful data for the development of high-throughput screening assays, as well as provisional insights into protein function without the isolation of the intact virus [46]. In addition to the fish viruses described here, we observed vertebrate host-associated viral transcripts from four other families (Figure 4). This indicates that with deeper sequencing of our libraries, and the use of rRNA depletion rather than poly-A enrichment, more viral data may have been recoverable, allowing for further taxonomic characterisation.

Notably, the viromes of freshwater fish from lacustrine and diadromous populations differed in their composition (Figure 5 and Figure 6). Specifically, the alpha diversity of vertebrate host-associated viruses of lacustrine *R. retropinna* was greater than that of diadromous individuals. Conversely, the alpha diversity of non-vertebrate host-associated viruses in diadromous *R. retropinna* was greater than that of lacustrine fish. This suggests that diadromous *R. retropinna* occupying coastal oceans and rivers may be infected by fewer fish viruses, but simultaneously, more non-vertebrate host-associated viruses. This may be the result of differences in viral diversity present in the surrounding marine environment, as a function of the overall species richness, which would be far less in lacustrine habitats. However, this trend was not observed in *G. cotidianus*. In this case, *G. cotidianus* individuals occupied a different aquatic niche than *R. retropinna* (see later) and therefore different viral ecology may be at play. In addition, our analysis of sample dispersion indicated that lacustrine and diadromous populations had significantly different taxonomic compositions of vertebrate host-associated viromes (Figure 6b), in turn suggesting that fish in diadromous and lacustrine populations differ in viral exposure.

Overall, *G. cotidianus* and *R. retropinna* vertebrate host-associated viromes varied significantly in their composition (Figure 5a,c and Figure 6a). This difference might relate to differences in the niches occupied by these fish species. For example, *G. cotidianus* is a benthic fish that lives and feeds on the bottom of aquatic ecosystems, while *R. retropinna* is a pelagic fish living in the water column [11,12]. It is possible that virions may concentrate in the benthic zone of aquatic ecosystems due to prevention of diffusion through the water column by the substrate, or because of decreased mixing. While far from conclusive, our results indicate a need for future studies to investigate the differences in viromes of pelagic and benthic fish to better understand how these differing niches may influence viral exposure.

Finally, we observed Toitoi astrovirus 1 in the lacustrine Lake Hayes and Lake Wanaka populations as well as the diadromous Otokaia Creek population of *G. cotidianus* (Figure 1a and Figure 4). Lake Hayes and Lake Wanaka are approximately 40 km apart (separated by The Crown Range), and both approximately 160 km from Otokaia Creek (with no direct hydrological connection). These three populations are geographically and hydrologically separated, yet the same astrovirus virus species, Toitoi astrovirus 1, was observed in the gill-tissue meta-transcriptomes of fish from each location (99.3–100% amino acid identity). Observing the same virus in these isolated populations may be indicative of viral transmission between these locations, presumably via some unknown vector although human interference via fish translocations cannot be ruled out. Sampling of piscivorous birds from overlapping fish habitats may shed light on vectors responsible for viral transmission over these geographical distances.

## Data Availability

All viral transcripts identified in this study are available on GenBank under accessions (OL871126—OL871169).

## Supplementary Materials

The following are available online at https://www.mdpi.com/article/10.3390/v14020257/s1: Table S1: Novel Fish Viruses of *Retropinna retropinna* and *Gobiomorphus cotidianus.* Table S2: Standardised Vertebrate-Associated Viral Abundance and Alpha Diversity. Figure S1: Non-vertebrate host-associated viral phylogenies. Figure S2: Standardised RPS13 reads of diadromous and lacustrine *R. retropinna* and *G. cotadiana* gill-tissue meta-transcriptomes. Figure S3: Regression analysis of Fulton’s condition factor and viral alpha diversity.
