# Supplementary material for: Viromes of Freshwater Fish with Lacustrine and Diadromous Life Histories Differ in Composition
Source: Viruses. 2022 Jan 27;14(2):257. doi: 10.3390/v14020257 (PMC8878276; doi:10.3390/v14020257)
Supplement: Supplementary file 1 [file viruses-14-00257-s001.zip › Supplement/Figure S1 Invertebrate Virus Phylogenies.pdf]

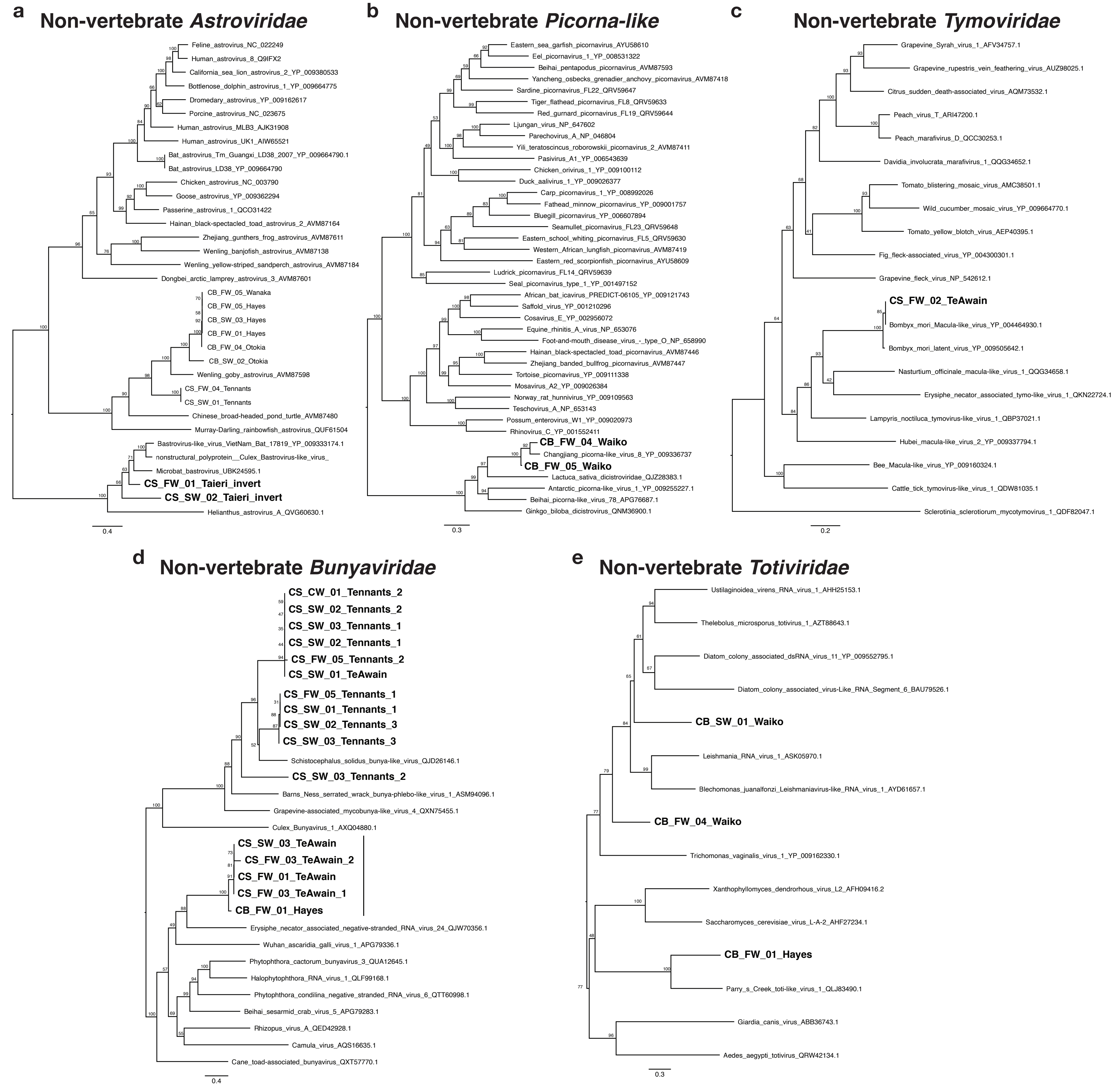
a
Non-vertebrate Astroviridae
b
Non-vertebrate Picorna-like
c
Non-vertebrate Tymoviridae
d
Non-vertebrate Bunyaviridae
e
Non-vertebrate Totiviridae
CS_FW_01_Taieri_invert
CS_SW_02_Taieri_invert
CB_FW_04_Waiko
CB_FW_05_Waiko
CS_FW_02_TeAwain
CS_CW_01_Tennants_2
CS_SW_02_Tennants_2
CS_SW_03_Tennants_1
CS_SW_02_Tennants_1
CS_FW_05_Tennants_2
CS_SW_01_TeAwain
CS_FW_05_Tennants_1
CS_SW_01_Tennants_1
CS_SW_02_Tennants_3
CS_SW_03_Tennants_3
CS_SW_03_Tennants_2
CS_SW_03_TeAwain
CS_FW_03_TeAwain_2
CS_FW_01_TeAwain
CS_FW_03_TeAwain_1
CB_FW_01_Hayes
CB_SW_01_Waiko
CB_FW_04_Waiko
CB_FW_01_Hayes
