# Supplementary figures and images for: Viromes of Freshwater Fish with Lacustrine and Diadromous Life Histories Differ in Composition
Source: Viruses. 2022 Jan 27;14(2):257. doi: 10.3390/v14020257 (PMC8878276; doi:10.3390/v14020257)

Standardized RPS13 Abundance

7e-04  
6e-04  
5e-04  
4e-04  
3e-04

*G. cotidianus*

*R. retropinna*

\*\*\*

\*\*\*

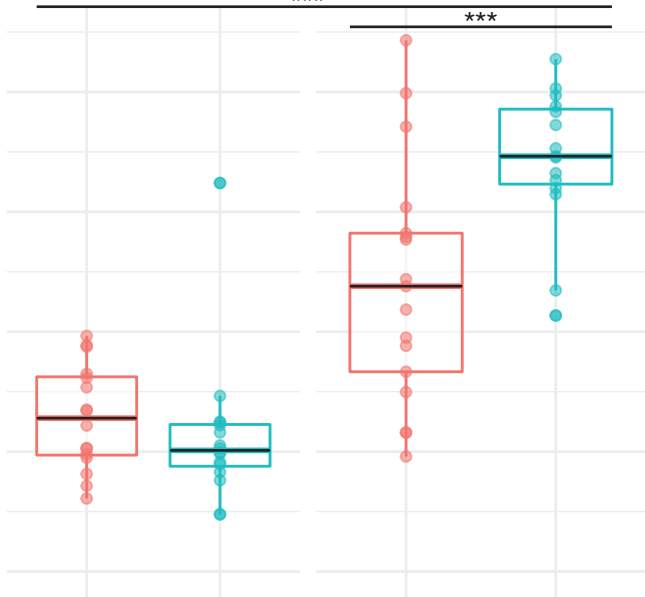

Supplement: Supplementary file 1 [file viruses-14-00257-s001.zip › Supplement/Figure S2 RPS13 Abundance.pdf]

## Diadromous

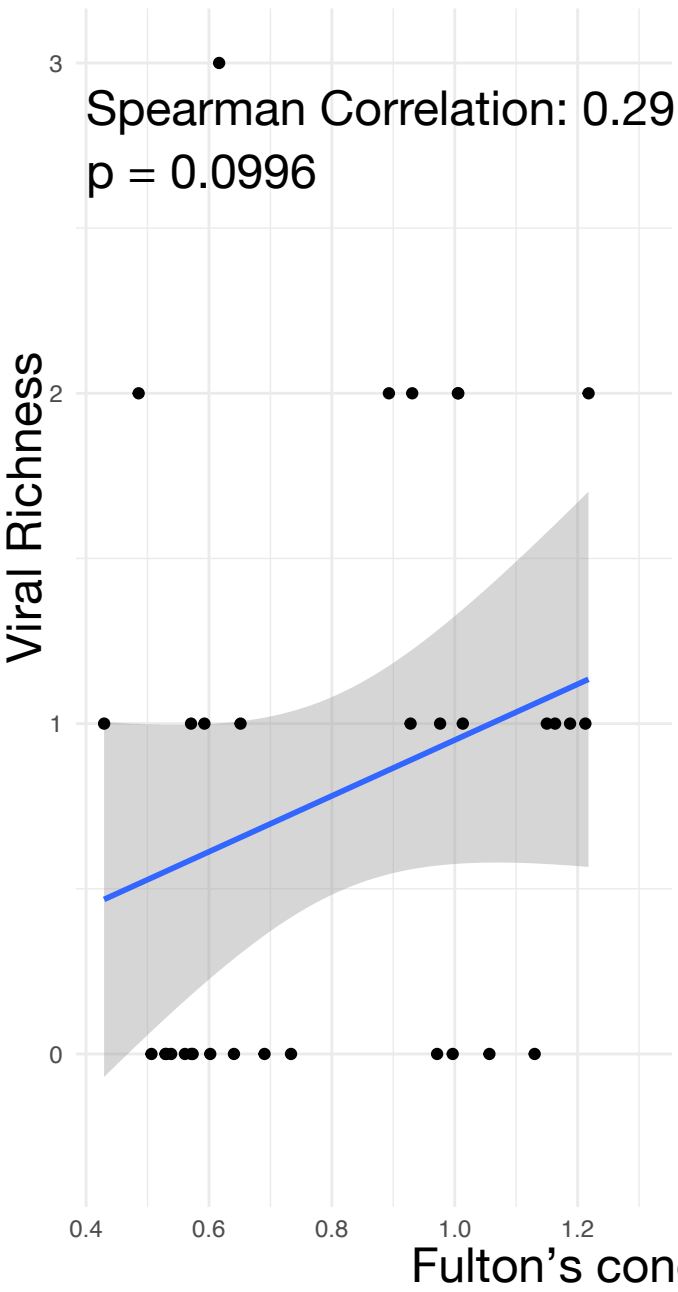

## Lacustrine

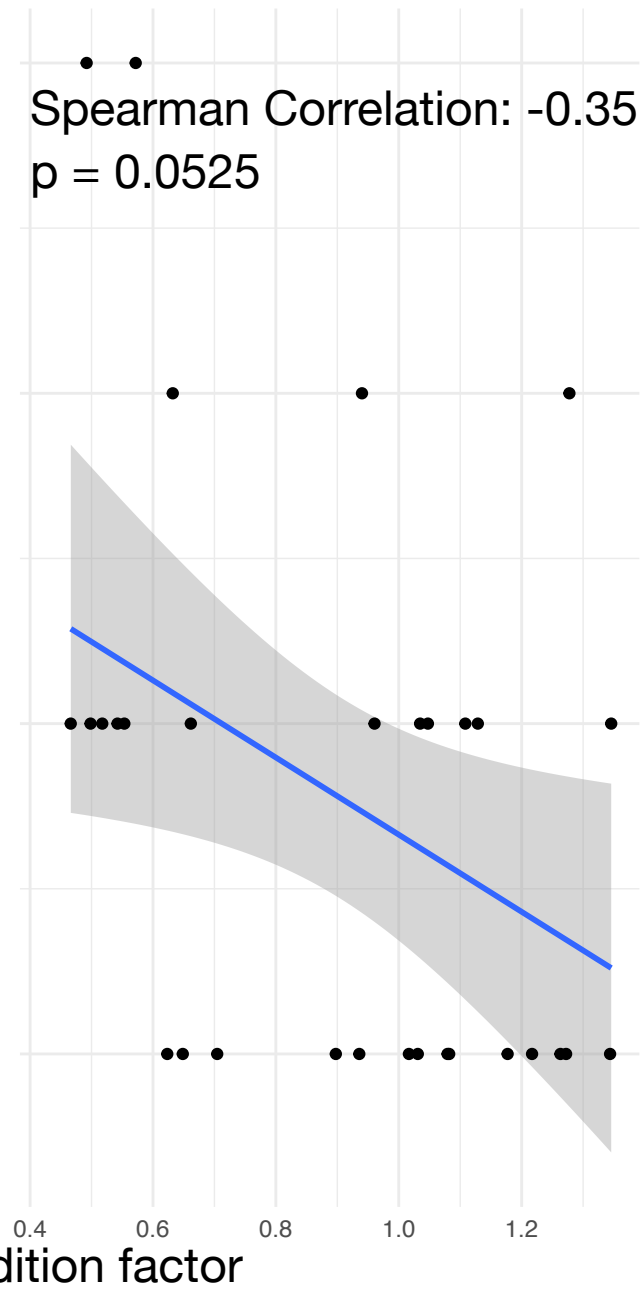

Supplement: Supplementary file 1 [file viruses-14-00257-s001.zip › Supplement/Figure S3 Body Condition Correlations.pdf]
